# Parathyroid adenoma presenting as chronic pancreatitis: A case report and literature review

**DOI:** 10.1097/MD.0000000000031750

**Published:** 2022-11-18

**Authors:** Chih-Hsuan Fu, Hua-Fen Chen

**Affiliations:** a Division of Endocrinology and Metabolism, Department of Internal Medicine, Far Eastern Memorial Hospital, New Taipei City, Taiwan, R.O.C; b Division of Hospital Medicine, Department of Internal Medicine, Far Eastern Memorial Hospital, New Taipei City, Taiwan, R.O.C; c School of Medicine and Department of Public Health, College of Medicine, Fujen Catholic University, New Taipei City, Taiwan. R.O.C.

**Keywords:** chronic pancreatitis, hyperparathyroidism, parathyroid adenoma

## Abstract

Most patients with parathyroid adenomas are asymptomatic and rarely present with chronic pancreatitis (CP). Several studies have reported a positive association between primary hyperparathyroidism (PHPT) and pancreatitis. Parathyroidectomy is the definitive treatment for PHPT. IV bisphosphonates can be considered the drug of choice for bridge to surgery.

**Methods:**

We reported a 57-year-old female patient was admitted to the emergency room with left upper quadrant abdominal pain and a diagnosis of recurrent pancreatitis. Magnetic Resonance Cholangiopancreatography confirmed the diagnosis of CP. The patient had no common etiology of pancreatitis. Persistent hypercalcemia was noted despite administering intravenous fluids, and Calcitonin. Intravenous Pamidronate, a Bisphosphonate derivative, was also administered. Although calcium levels initially decreased, they were later found to rebound to previous levels.

**Results:**

A diagnosis of parathyroid adenoma and PHPT was made based on the elevated parathyroid hormone levels and cervical ultrasonography indicated right inferior parathyroid adenoma. Technetium-99m methoxy-isobutyl-isonitrile scintigraphy revealed a focal hot spot of tracer accumulation at the right lower thyroid bed. The patient underwent right lower parathyroidectomy smoothly and successfully. After right lower parathyroidectomy, she had normal serum calcium levels (9.2 mg/dL) and parathyroid hormone (16.1 pg/mL). There was no recurrent abdominal pain after the operation.

**Conclusion:**

CP is a rare manifestation of parathyroid adenoma. When patients with a history of recurrent pancreatitis, without common causes of pancreatitis, present persistent elevated serum calcium levels, PHPT could be suspected.

## 1. Introduction

A parathyroid adenoma is a benign tumor of the parathyroid glands that it is generally responsible for 80% to 85% cases of hyperparathyroidism.^[1]^ Patients with primary hyperparathyroidism (PHPT) are usually asymptomatic. In contrast, symptomatic patients usually exhibit symptoms of hypercalcemia, such as painful bones, kidney stones, and abdominal and psychic groans,^[2]^ whereas chronic pancreatitis (CP) is a rare clinical manifestation. Herein, we report on a case of a 57-year-old woman without any common risk factor for pancreatitis, who was admitted to our ward with a diagnosis of CP. Further workup revealed PHPT, due to a functioning parathyroid adenoma. In addition, we performed a literature review to further explore and assess the relationship between PHPT and pancreatitis.

## 2. Case presentation

A 57-year-old female patient was presented to the emergency room with left upper quadrant abdominal pain described as sharp, unremitting and radiating to the back for 4 days prior to admission. She had been hospitalized for acute pancreatitis (AP) with unknown causes 3 times during the past 1 year and had undergone extracorporeal shock wave lithotripsy 2 times due to symptomatic bilateral nephrolithiasis and left ureterolithiasis. The patient had a history of type 2 diabetes mellitus receiving Metformin 1000 mg per day, and hypertension receiving Bisoprolol 5 mg twice a day and 1 tablet of Coaprovel per day, which is a combination of irbesartan 300 mg plus hydrochlorothiazide 12.5 mg prescribed by a general physician. She was a never-smoker and never-drinker.

On initial examination, her temperature was 36.2°C, blood pressure 93/60 mm Hg, heart and respiratory rate were 65 beats per minute and 18 breaths per minute, respectively. Oxygen saturation was 99% while breathing ambient air. The bowel sounds were hypoactive, and diffuse abdominal tenderness was present without rebound, guarding, or ascites. Otherwise, the patient’s physical examination was unremarkable. A hemogram revealed no leukocytosis 9.44 × 10 3/μL (reference range:3.80-10.40 × 10 3/μL) and a normocytic anemia of hemoglobin 10.9 g/dL (reference range: 12.0–16.0 g/dL) with mean corpuscular volume of 90.9 fL (reference range: 82.0–101.0 fL). Normal renal function of Creatinine 1.18 mg/dL (reference range: 0.60–1.20 mg/dL) was found. A significantly elevated lipase of 1041 U/L (reference range: 11–82 U/L) with liver transaminases, total bilirubin, alkaline phosphatase and gamma-glutamyl-transferase within normal ranges were noted. A tentative diagnosis of recurrent pancreatitis was made based on the patient’s history, clinical presentation and laboratory findings.

**Figure 1.**
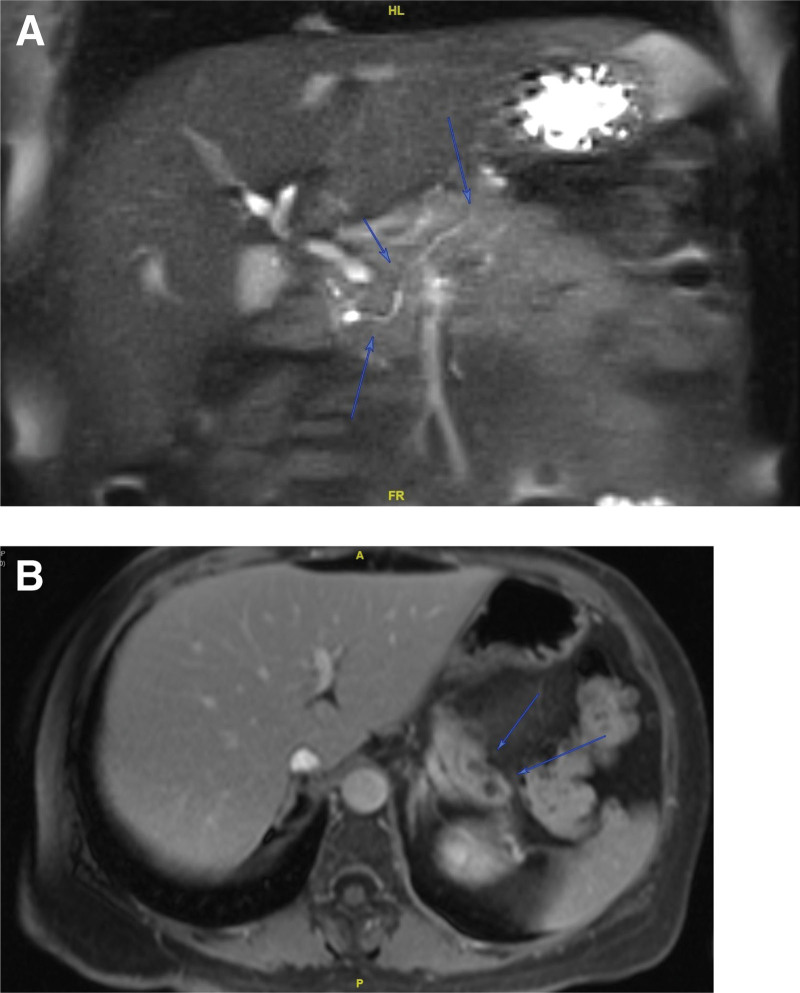
MRCP of the abdomen showing main pancreatic duct irregularities (A) and atrophy of the pancreas with 2 discrete small pseudocysts in the more contracted tail region (B). MRCP = magnetic resonance cholangiopancreatography.

To investigate the etiology of pancreatitis, ultrasound of the abdomen showed mild fatty liver and a dilated common bile duct measuring 0.9 cm in diameter. Magnetic Resonance Cholangiopancreatography revealed main pancreatic duct irregularities (Fig. 1A) and atrophy of the pancreas with 2 discrete small pseudocysts in the more contracted tail region (Fig. 1B). Further blood testing revealed normal ANA, IgG4 and triglyceride. Also, results showed elevated serum calcium levels (hypercalcemia) of 11.9 mg/dL (reference range: 8.6–10.3 mg/dL) and normal albumin levels.

**Figure 2.**
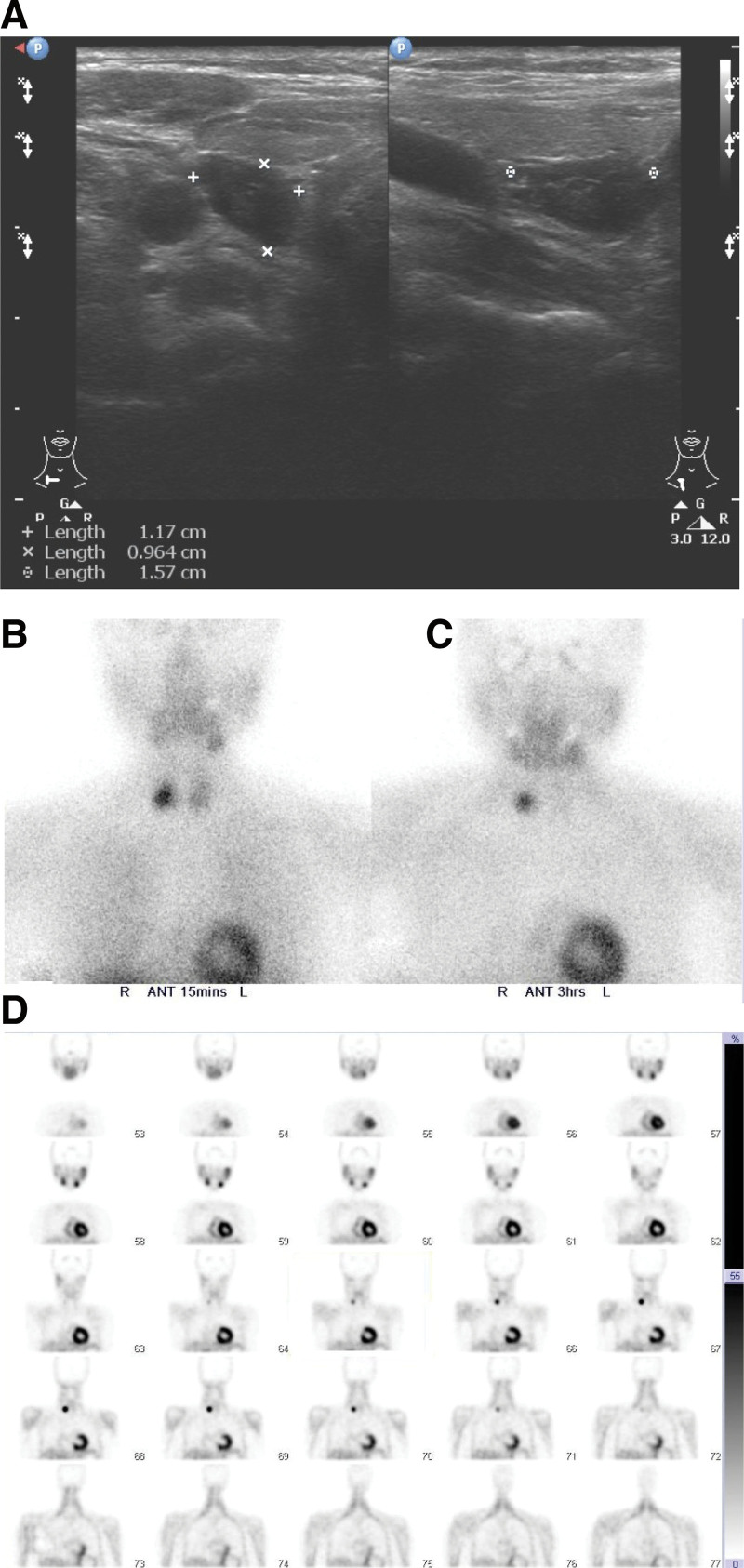
Cervical ultrasonography showing a heterogeneous hypoechoic well-defined mass in the right thyroid bed measuring 1.17 × 0.96 × 1.57 cm in maximal dimension. (A); Tc-99m MIBI showing a focal hot spot of tracer accumulation at the right lower thyroid bed at 15 minutes and (B); at 3 hours delayed phase (C); SPECT did not show definite abnormal foci of tracer activity in the thorax (D). Tc-99m MIBI = Technetium-99m methoxy-isobutyl-isonitrile scintigraphy, SPECT = Sestamibi-single photon emission computed tomography.

Initial patient management included fasting, intravenous fluids, electrolyte repletion, and analgesics. Her abdominal pain improved gradually and lipase levels also decreased. We managed to discontinue hydrochlorothiazide, ensured adequate hydration and prescribed Calcitonin for hypercalcemia. However, her calcium levels remained high, thus we prescribed intravenous Pamidronate, a Bisphosphonate derivative. Consequently, calcium levels decreased temporarily but rebounded after 72 hours. Elevated intact parathyroid hormone (iPTH) of 107.8 pg/mL (reference range: 8.0–76 pg/mL) was noted and cervical ultrasonography revealed the presence of a heterogeneous hypoechoic well-defined mass in the right thyroid bed (Fig. 2A). Technetium-99m methoxy-isobutyl-isonitrile scintigraphy (Tc-99m MIBI) showed a focal hot spot of tracer accumulation at the right lower thyroid bed, that was highly suspicious for parathyroid adenoma (Fig. 2B and C) and Sestamibi-single photon emission computed tomography (SPECT) showing no definite abnormal foci of tracer activity in the thorax (Fig. 2D). Finally, the diagnosis of hypercalcemia-induced CP due to PHPT was made.

**Figure 3.**
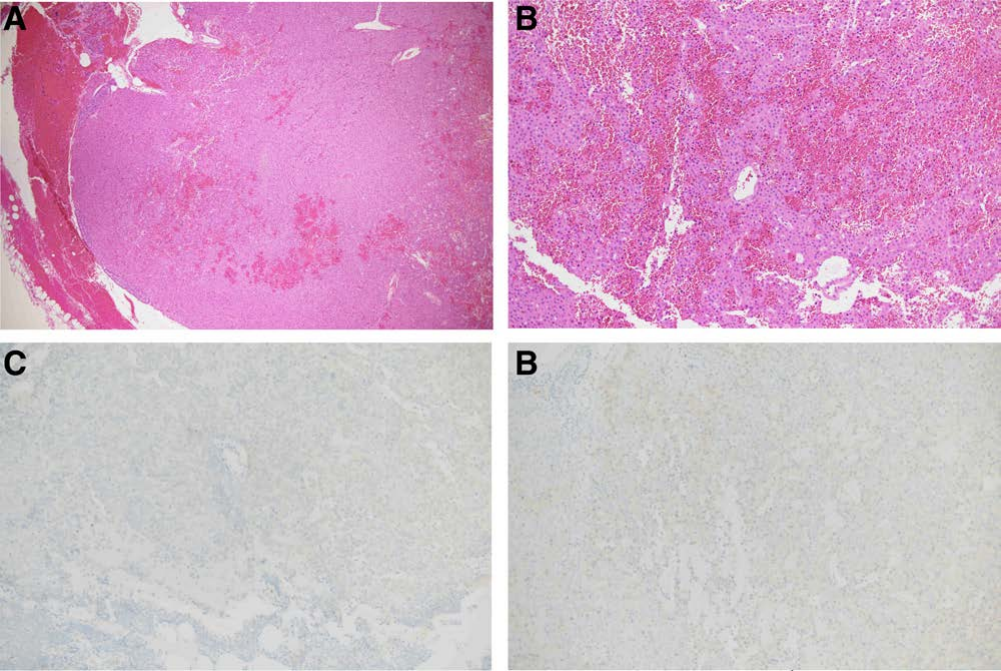
Most parathyroid adenomas consist of chief cells; Hematoxylin – eosin stained sections of the adenoma (A–B); TTF-1-negative neoplastic cells without a thick fibrous capsule (C); Galectin-3 stain was negative (D). (A: 40 × original magnification; (B–D): 100 × original magnification).

The patient underwent right lower parathyroidectomy under general anesthesia. Histological examination was consistent with the diagnosis of parathyroid adenoma (Fig. 3). The patient was discharged on the third postoperative day with her serum calcium levels maintained at 9.2 mg/dL and PTH at 16.1 pg/mL. Six months after successful parathyroid surgery, there no recurrence of abdominal pain has been noted.

## 3. Discussion

The first reported case of AP related to hyperparathyroidism was reported as early as 1940 by Smith and Cooke^[3]^ and in 1966, Pyrah et al^[4]^ attempted to summarize the circumstances under which pancreatic disease occurred in association with PHPT. In 2006, Jacob et al^[5]^ classified the presentation of pancreatic disease in PHPT into 4 classes: PHPT presenting as AP; PHPT presenting as recurrent AP with no evidence of CP; PHPT presenting as CP with or without pancreatic calcification; and PHPT complicated by AP in the postoperative period. Meanwhile, PHPT was accepted as 1 of the etiologies for AP.^[6]^

The relationship between PHPT and pancreatitis remains controversial. Bess et al^[7]^ found that 1.5% of patients with surgically confirmed PHPT, had coexisting or prior pancreatitis, whereas 64.7% of the patients had at least 1 concomitant etiology for pancreatitis, such as gallstones or alcohol abuse and symptoms due to pancreatitis did not improve after hyperparathyroidism treatment. Our case suggests a true positive causal association between PHPT and pancreatitis for there were no additional risk factors for pancreatitis. Khoo et al^[8]^ noted that only 1.5% of community patients with PHPT had AP, which is similar to the frequency of AP observed in the matched control group. It should be noted that this study was a community-based PHPT cohort, unlike other surgical- or hospital-based cohorts, with patients having more severe hypercalcemia.

Nowadays several studies have reported a positive association between PHPT and pancreatitis. The majority of patients (approximately 70% of all cases) presenting with pancreatic disease in PHPT have concurrent AP, while patients with CP are approximately 30%.^[9]^ Diallo et al^[10]^ reported that 5 of 61 pancreatitis cases had PHPT, of which 2 patients presented with CP and the prevalence of PHPT-associated CP was approximately 3.27% of all hospitalized cases for pancreatitis. Three subsequent studies from India also reported a low prevalence of PHPT-associated CP. Aslam et al^[11]^ reported that 1.94% of pancreatitis cases (77 out of 3962) had PHPT, of which 36 patients presented as CP and the prevalence of PHPT-associated CP was approximately 0.9% of all hospitalized cases for pancreatitis. Furthermore, Thareja et al^[12]^ reported that 9.4% of pancreatitis cases (5 out of 70) had PHPT, of which 1 patient presented as CP and the prevalence of PHPT-associated CP was approximately 1.42% of all hospitalized cases for pancreatitis. The latest study^[13]^ analyzed 242 patients with PHPT and found that 6.19% of PHPT cases (15 out of 242) had pancreatitis, of which 1 patient had chronic calcific pancreatitis and the prevalence of CP was approximately 0.41% of all PHPT cases. The authors concluded that pancreatitis can be the only presenting complaint of PHPT.

The different etiology of CP may have different clinical characters. Bhadada et al^[14]^ compared the clinical profile of CP related to PHPT with other forms of CP such as CP related to alcohol and idiopathic CP. The male to female ratio was significantly greater in CP related to alcohol (P, = 0.005). Renal colic, nephrolithiasis, and nephrocalcinosis were significantly more common in the CP related to PHPT group compared with other groups (*P* = .000, .000, and .018, respectively). Biochemical parameters revealed that the mean corrected calcium level was significantly higher in the PHPT with CP group than other groups (*P* = .001) and the mean serum phosphate level was significantly lower in the PHPT with CP group compared with other groups (*P* = .04). This finding is probably due to the action of PTH, which facilitates increased renal excretion of phosphorous. Other biochemical parameters such as serum amylase, serum alkaline phosphatase, blood sugar, and triglyceride levels were not different across the 3 CP groups. The mean iPTH level was significantly higher in the PHPT with the CP group compared with other groups (*P* = .009). However, the serum iPTH level was significantly higher in the PHPT without CP group (*P* = .004) when PHPT patients without CP were compared with patients with CP. Misgar et al^[13]^ also found that a higher iPTH level was noted in PHPT patients with pancreatitis; however, this finding was not statistically significant (*P* = .225). This can be explained by the fact that patients suffering from PHPT with pancreatitis usually complaint of intense abdominal pain and thus could be diagnosed earlier than patients suffering from PHPT without pancreatitis. In PHPT patients, therefore, serum iPTH levels have no obvious relationship with the incidence of pancreatitis.

The pathophysiology of CP is complex and differs by etiology. Although the definite mechanism of PHPT-induced CP is not well understood, it is believed that sustained hypercalcemia from uncontrolled PHPT leads to premature pancreatic protease activation followed by pancreatic acinar cell damage.^[15]^ Repeated episodes of AP will create a chronic inflammation environment, which in turn activates the pancreatic stellate cells that are major mediators of fibrosis.^[16]^

The diagnosis of PHPT-induced CP is made based on the patient’s history, clinical presentation, biochemical studies and imaging findings. Patients may present with recurrent episodes of AP. Elevation of iPTH and calcium level and decrease in phosphate levels indicate PHPT.

Imaging modalities can help localize but also identify patients who are candidates for a minimally invasive parathyroidectomy (MIP) for single gland disease or bilateral neck exploration for multigland diseases and negative imaging results.^[17]^ Cervical ultrasonography is widely used as the initial localization study in patients with PHPT, because it is a low-cost, safe, without radiation exposure and highly sensitive technique.^[18]^ Normal parathyroid glands have the same echogenicity as the thyroid and thus usually remain unidentified on ultrasonography. Parathyroid adenoma is typically homogenous, hypoechoic, circumscribed, and oval-shaped. On color Doppler, adenoma may demonstrate a polar feeding vessel with peripheral vascularity, which is known as the “vascular arch,” that helps differentiate this benign tumor from lymph nodes or tumor lesions, whose vascularization would be more central.^[19]^ Tc-99m MIBI with SPECT of the neck can provide additional functional information and is more sensitive for ectopic adenomas that are either undetected or missed by ultrasound, especially in the mediastinal location and deep/posterior glands.^[17]^

The range of sensitivities and specificities of cervical ultrasonography and Tc-99m MIBI reported in literature varies widely. A meta-analysis^[20]^ revealed a pooled sensitivity of 80% for cervical ultrasonography and 83% for scintigraphy, and a pooled specificity of 77% for cervical ultrasonography and 87% for scintigraphy. Concurrent use of these 2 modalities can improve sensitivity, rendering this combination the most sensitive method of localizing parathyroid adenoma in patients with hyperparathyroidism.^[21]^ Furthermore, both modalities have widespread availability and no risks of contrast allergy or renal failure. Now, combination of cervical ultrasound and Tc-99m MIBI SPECT is an acceptable first-line strategy for preoperative imaging localization.^[22]^

Parathyroidectomy remains the only definitive treatment of PHPT and is more cost-effective than long-term observation or pharmacologic therapy.^[23]^ A systemic review^[24]^ including a total of 5282 patients from 14 studies found that the long-term (≧1 year) cure rate, defined as normocalcemia persisting for more than 6 months after MIP was 96.9%. The overall complication rate was 4.4% and transient postoperative hypocalcemia was the most common complication. Medical management can be considered in patients with PHPT who refuse parathyroidectomy and do not meet surgical criteria for intervention. Alendronate, the most widely used oral bisphosphonate, can improve bone marrow density and can also be used as a bridge to surgery.^[25]^ IV bisphosphonates such as Pamidronate and Zoledronate are more potent than oral bisphosphonates and have been approved for use in the treatment of hypercalcemia and osteoporosis. Adequate hydration is crucial when treating hypercalcemia in hospitalized patients, and Calcitonin can transiently reduce the serum calcium concentration by inhibiting bone resorption and promoting urinary calcium excretion. Intravenous Pamidronate may also be used for treating hyperparathyroidism-related hypercalcemia^[26]^ and can be considered another drug of choice for bridge to surgery. In our case, hypercalcemia persisted after adequate hydration and administration of Calcitonin. Although Pamidronate reduced calcium level temporarily, this drug earns more time to prepare for operation. There has been no postoperative hypocalcemia 6 months after successful MIP and no recurrence of abdominal pain.

## 4. Conclusions

CP is a rare manifestation of primary adenoma. When patients with CP have no common etiology defined and are associated with elevated serum calcium, PHPT-induced CP should be suspected. Parathyroidectomy is a safe, effective, and definitive treatment for parathyroid adenoma. Early recognition is the best way to deal with this condition.

## Author contributions

**Writing – original draft:** Chih-Hsuan Fu.

**Writing – review & editing:** Hua-Fen Chen.
